# Insight into the Dynamics of Fractional Maxwell Nano-Fluids Subject to Entropy Generation, Lorentz Force and Heat Source via Finite Difference Scheme

**DOI:** 10.3390/nano12101745

**Published:** 2022-05-20

**Authors:** Muhammad Imran Asjad, Muhammad Usman, Arfan Ali, Jan Awrejcewicz, Maksymilian Bednarek

**Affiliations:** 1 Department of Mathematics, University of Management and Technology, Lahore 54770, Pakistan; arfanali347@gmail.com; 2 Department of Mathematics, National University of Modern Languages (NUML), Islamabad 44000, Pakistan; dr.usman@numl.edu.pk; 3 Department of Automation, Biomechanics, and Mechatronics, Faculty of Mechanical Engineering, Lodz University of Technology, 90-924 Lodz, Poland; jan.awrejcewicz@p.lodz.pl (J.A.); maksymilian.bednarek@p.lodz.pl (M.B.)

**Keywords:** fractional order, Maxwell fluids, nano-fluids, entropy generation (EG), Bejan number

## Abstract

In recent times, the loss of useful energy and solutions to those energy challenges have a wide scope in different areas of engineering. This work focuses on entropy analysis for unsteady viscoelastic fluids. The momentum boundary layer and thermal boundary layer are described under the effects of a magnetic field in the absence of an induced magnetic field. The study of a fractional model of Maxwell nanofluid by partial differential equation using Caputo time differential operator can well address the memory effect. Using transformations, the fractional ordered partial differential equations (PDEs) are transfigured into dimensionless PDEs. Numerical results for fractional Maxwell nanofluids flow and heat transfer are driven graphically. The Bejan number is obtained following the suggested transformation of dimensionless quantities like entropy generation. A mathematical model of entropy generation, Bejan number, Nusselt number and skin friction are developed for nanofluids. Effects of different physical parameters like Brickman number, Prandtl number, Grashof number and Hartmann number are illustrated graphically by MAPLE. Results depict that the addition of nanoparticles in base-fluid controls the entropy generation that enhances the thermal conductivity and application of magnetic field has strong effects on the heat transfer of fractional Maxwell fluids. An increasing behavior in entropy generation is noticed in the presence of source term and thermal radiation parameter.

## 1. Introduction

The best way to represent the natural phenomena is by using differential equations (DEs) with suitable boundary conditions. Recently, the fractional (non-integral) order of DEs has gained much interest because of their vast scope in many engineering fields like food engineering, oceanography, chemical reactions and chaos. All fractional derivatives like Riemann Levillie, Caputo, Caputo Fabrizio, Antangna Beleanu are used widely in practice [1,2,3], as a deep study can easily be handled using this approach. Natural convection along a vertical wall and cylinder has been explained using Caputo time-fractional derivatives and fractional derivatives [4,5]. The significant difference between fractional and ordinary fluid flow emerges at different times. Furthermore, with large time values, increasing the fractional parameter’s value increases velocity. Fractional calculus covers the complex structure of viscoelastic fluids in various research areas of glass fiber production, exotic lubricants, colloidal solutions, extraction of polymer solutions and cooling processes [6]. A simplified Phan–Thien–Tanner model for viscoelastic fluids was investigated analytically in [7] with different physical parameters. It was established by Sheikh, N.A. et al. [8] that it is not enough to get experimental data by using a conventional derivative model for Maxwell fluids rather than fractional operators. Moreover, it was discovered that a link exists between Maxwell’s constitutive equation and molecular theory [9]. Few other investigations for analytical results have been done in many research articles. But in the rheological perspective, viscoelastic fractional order models have attracted much interest due to their wide range of applications. Fractional Maxwell models are derived by substituting the conventional derivative in the known Maxwell model of stress-strain expression by fractional-order derivatives. Analytical solutions of fractional Maxwell, fractional generalized Maxwell model, fractional second grade, third grade, Oldroyd-B model etc., via analytical techniques of Laplace transform, Fourier transform, Weber transform and Hankel transform are obtained in [10,11,12,13,14].

Many researchers focus on such physical processes involving entropy generation-EG. It is a well-known fact that all physical problems, especially heat transfer, involve entropy generation—EG. Entropy generation-EG plays a vital role in fluid dynamics. The first law of thermodynamics moves around heat transfer processes, whereas the second law of thermodynamics moves around the entropy generation of the system. Entropy generation—EG tells the feasibility and efficiency of the system. In other words, entropy describes the ways a process can control energy loss.

Since entropy generation—EG is important and happens in almost all thermo-dynamical processes, many researchers have worked in this direction. Among the various researchers who have done splendid work on entropy generation–EG, Adrian Bejan, has published many articles and books [15,16,17,18,19,20,21]. Considerable work is done by Pranab Kumar Mondal [22] for irreversibility analysis of Couette flow while applying weak and relatively strong pressure gradient. The fluid dynamics are studied by variation of volumetric entropy generation number and Bejan number.

To control the entropy generation—EG, addition of nanoparticles in base fluid gave a new direction to heat transfer problems (the formation of nanofluids). Nanofluids are nano-sized particles accomplished with base-fluids, i.e., Common nanoparticles Cu, Ag, Au and Fe. Water, engine oil and ethylene glycol are common base fluids. To enhance the thermal conductivity of base fluids, the idea of nanofluids is given by Choi et al. in [23] for the first time. Later on, Tiwari and Das discussed the effects of different shapes of nano-sized particles on thermal enhancement [24]. Using square shape cavity, different aspects of ball-shaped, cylindrical-shaped, and rod-shaped nanoparticles were studied [25]. Analytical results for the effects of nanofluids were driven by [26]. In [27], analytical results are investigated for temperature profile and Nusselt number under the effects of viscous dissipation and porous media. Using a traditional approach, numerical results were obtained, expressing the radial and tangential momentum across the disk decreases for higher Lorentz forces and slip factor [28].

Like many other key sources, entropy generation—EG includes viscous dissipation, chemical reaction, heat and mass transfer, heat convection and conduction and electrical conduction. Considerable work by B. Mahathish et al. has been done via the spectral quasi-linearization (SQL) method for entropy analysis and solving a Williamson model, which provides a base for fractional models. An analytical approach is applied to investigate the effects of viscous dissipation and limiting effects of Nusselt for temperature profile [29]. Bejan number-BN is the ratio of entropy generation—EG due to heat and total entropy generation—EG of system. But Awed in [30] gave a new definition to Bejan number-BN. Bejan number-BN describes the effects of magnetic field irreversibility and fluid friction irreversibility. An investigation has been conducted on laminar falling liquid film along an inclined heated surface in [31]. In [32,33,34] and many other articles, the exact and numerical solution of entropy generation—EG is published. B. Mahathish numerically investigates the effects of quadratic variation of density-temperature (quadratic convection) and the quadratic Rosseland thermal radiation using the modified Bongiorno Model (MBM) [35]. Similar analytical as well as numerical approaches can be seen in [36,37,38,39] for such different viscoelastic models.

Numerical investigations and many others were used in [40,41,42,43]; however, various research gaps are found in these articles, which are still not addressed. Such as how heat transfer can be enhanced by adding the nanoparticle to the base fluid? What is the effect of Lorentz forces on flow dynamics? What do numerical results predict about Skin friction and Nusselt number? What are the formulation of entropy generation and Bejan number in the presence of thermo-physical properties of nanoparticles? In reviewing these research gaps in literature surveys, the main task of this article is to develop a mathematical model of momentum and heat for fractional Maxwell nano-fluids for detailed insights into Lorentz force, heat source/sink, and Nusselt number and entropy generation—EG. A new definition of Bejan number—BN is introduced by adding the coefficients of thermo-physical properties of nanoparticles. Water is taken as base fluid, whereas $Cu\;\mathrm{and}\;Al_{2}O_{3}$ are the nanoparticles used for graphical results of velocity and temperature profiles. The problem is first modeled fractionally by applying the definition of Caputo time derivative, then using transformation; a dimensionless analysis is done. The resulted model is solved by a numerical technique of finite difference scheme. Plots are drawn for Bejan number—BN against Br the Brickman number, $H_{a}$ the square of Hartmann number and Ω dimensionless temperature difference. Moreover, some graphical results are extended to evaluate the Nusselt number Nu and Skin friction $S_{f}$. These results are computed via mathematical software MAPLE.

## 2. Mathematical Model and Formulations

**Figure 1:**
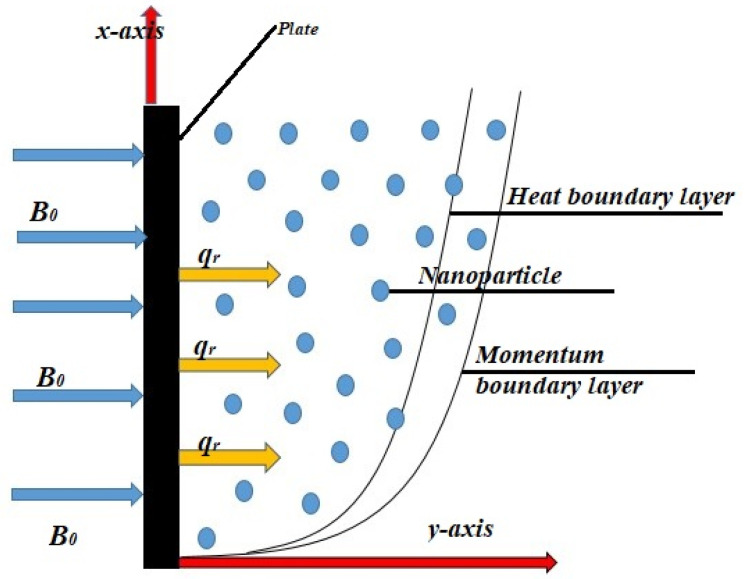
Geometry of the problem.

Considering magnetohydrodynamic (MHD) flow of incompressible and unsteady fluid along the infinite vertical plate. The induced magnetic field and pressure gradient are neglected. Initially, at time t=0, fluid has velocity zero and has a constant temperature $\theta_{\infty }$. With the passage of time temperature of the system rise to $\theta_{w}$. The fluid flow is considered along x−direction. The magnetic field is applied in the y−direction as illustrated in Figure 1 below.

Taking into account the Boussinesq approximation, the assumptions of the system are,

- Flow is unsteady, incompressible and 1-dimensional.
- Pressure gradient is absent.
- Body force is significant.
- Magnetic field is applied (ignoring the induced magnetic field).

Then the equation of continuity is restricted and takes the following form (can be seen in [44]);

(1) ∇·V=0

But the Navier–Stoke equation [45,46] takes the form,

(2) $$\rho_{nf}\left[\frac{\partial V}{\partial t}+\left(V\cdot \nabla \right)V\right]=divT+g{\left(\rho \beta \right)}_{nf}\left(\theta -\theta_{\infty }\right)+J\times B$$

where $\rho_{nf},\;T,\;J,\;B,\;g,\;\beta_{nf},\;\theta \;\mathrm{and}\;\theta_{\infty }$ are dynamic viscosity of nanofluid, Cauchy stress tensor, current density, total magnetic field, gravitational acceleration, thermal expansion coefficient, the temperature of nanofluid and ambient temperature, respectively.

The stress tensor for Maxwell fluids in [47] as,

(3) T=−pI+S,

where

(4) $$S+\lambda_{1}\frac{\delta S}{\delta t}=\mu A_{1},$$

In these expressions $S,\;A_{1},\lambda_{1},\;\mu ,\;D/Dt$ represents the extra stress tensor, Rivline–Ericksen tensor, kinematic viscosity and material time derivative. Also $A_{1}$ is expressed in [48] as;

(5) $$A_{1}=\left(gradV\right)+{\left(gradV\right)}^{T},$$

And

(6) $$\frac{\delta S}{\delta t}=\frac{DS}{Dt}-LS-SL^{T},$$

By following the assumption of the problem, the Maxwell equation can be written in [49] as;

(7) $$J\times B=-\left(\;\sigma_{nf}B_{0}^{2}u,0,0\;\right),$$

Keep in view that $B=B_{0}+b_{0}$ is the sum of applied and induced magnetic field (neglected).

Using Equations (3)–(7), the Equation (2) takes the form;

(8) $$\rho_{nf}\frac{\partial u}{\partial t}=\frac{\partial S_{xy}}{\partial y}+g{\left(\rho \beta_{\theta }\right)}_{nf}\left(\theta -\theta_{\infty }\right)-\sigma_{nf}B_{0}^{2}u,$$

Multiplying both sides of Equation (8) by $\left(1+\lambda_{1}^{\alpha }D_{t}^{\alpha }\right)$

(9) $$\begin{array}{c} \left(1+\lambda_{1}^{\alpha }D_{t}^{\alpha }\right)\rho_{nf}\frac{\partial u}{\partial t}=\left(1+\lambda_{1}^{\alpha }D_{t}^{\alpha }\right)\frac{\partial S_{xy}}{\partial y}+g{\left(\rho \beta_{\theta }\right)}_{nf}\left(1+\lambda_{1}^{\alpha }D_{t}^{\alpha }\right)\left(\theta -\theta_{\infty }\right)\\ -\sigma_{nf}B_{0}^{2}\left(1+\lambda_{1}^{\alpha }D_{t}^{\alpha }\right)u. \end{array}$$

But the fractional constitutive equation for Maxwell fluids is given by [50],

(10) $$\left(1+\lambda_{1}^{\alpha }D_{t}^{\alpha }\right)S_{xy}=\mu \frac{\partial u}{\partial y}\;\mathrm{with}\;0<\alpha <1,$$

This constitutive relation contains $D_{t}^{\alpha }$, Caputo fractional operator is defined in [51] as;

(11) D0Ctαf(t)=1Γ(1−α)∫0t(t−η)−α∂f(η)∂ηdη, 0<α<1,

With Γ(·) as the Gamma function defined in [51] by;

(12) $$\Gamma \left(z\right)=\int \eta^{z-1}e^{-\eta }d\eta ,\;z\epsilon \mathbb{C},\;\mathbb{R}e\left(z\right)>0.$$

Using Equation (10) into Equation (9)

(13) $$\begin{array}{c} \left(1+\lambda_{1}^{\alpha }D_{t}^{\alpha }\right)\rho_{nf}\frac{\partial u}{\partial t}=\mu_{nf}\frac{\partial^{2}u}{\partial y^{2}}+\left(1+\lambda_{1}^{\alpha }D_{t}^{\alpha }\right)g{\left(\rho \beta_{\theta }\right)}_{nf}\left(\theta -\theta_{\infty }\right)\\ -\left(1+\lambda_{1}^{\alpha }D_{t}^{\alpha }\right)\sigma_{nf}B_{0}^{2}u, \end{array}$$

The first law of thermodynamics [46,52] is;

(14) $${\left(\rho C_{p}\right)}_{nf}\left(\frac{\partial \theta }{\partial t}\right)=K_{nf}\frac{\partial^{2}\theta }{\partial y^{2}}-\frac{\partial q_{r}}{\partial y}+Q\left(\theta -\theta_{\infty }\right).$$

In this equation ${\left(\rho C_{p}\right)}_{nf},\;K_{nf},\;q_{r}\;\mathrm{and}\;Q$ are constant of heat capacity, coefficient of thermal conductivity, radiative heat flux, and nanofluid thermal conductivity, respectively.

By using the Rosselands approximation for fluids that are considered optically thick, the radiative heat flux $q_{r}$ expressed in [53] is given as;

(15) $$q_{r}=-\frac{4\sigma^{*}}{3k^{*}}\frac{\partial \theta^{4}}{\partial y},$$

In this expression $\sigma^{*}\;\mathrm{and}\;k^{*}$ are the Stefan–Boltzmann constant and mean spectral absorption constant, respectively. Approximating $\theta^{4}$ by a Taylor’s series expansion in the neighborhood of $\theta_{\infty }$ and neglecting higher power.

$\theta^{4}=4\theta_{\infty }^{3}\theta -3\theta_{\infty ,}^{4}$ (neglecting higher power), so radiative heat flux is

(16) $$q_{r}=-\frac{16\sigma^{*}\theta_{\infty }^{3}}{3k^{*}}\frac{\partial \theta }{\partial y}.$$

Then Equation (12) becomes

(17) $${\left(\rho C_{p}\right)}_{nf}\left(\frac{\partial \theta }{\partial t}\right)=K_{nf}\frac{\partial^{2}\theta }{\partial y^{2}}\left(1+\frac{16\sigma^{*}\theta_{\infty }^{3}}{3k^{*}K_{nf}}\right)+Q\left(\theta -\theta_{\infty }\right).$$

The second law of thermodynamics is given by [46],

(18) $$E_{G}=\frac{K_{nf}}{\theta_{\infty }^{2}}\left(1+\frac{16\sigma^{*}\theta_{\infty }^{3}}{3k^{*}K_{nf}}\right){\left(\frac{\partial \theta }{\partial y}\right)}^{2}+\frac{\mu_{nf}}{\theta_{\infty }}{\left(\frac{\partial u}{\partial y}\right)}^{2}+\frac{\sigma_{nf}B_{0}^{2}}{\theta_{\infty }}u^{2}.$$

In which $E_{G}\;\mathrm{and}\;\sigma_{nf}$ are volumetric local entropy generation and electrical conductivity.

The proposed boundary and initial conditions of this physical phenomenon are defined in [54] below;

(19) $$\begin{array}{c} u\left(y,0\right)=0,\;u_{t}\left(y,0\right)=0,u\left(0,t\;\right)=u_{0}e^{at},u\left(\infty ,t\right)=0\\ \theta \left(y,0\right)=\theta_{\infty },\theta \left(0,t\right)=\theta_{w},\;\theta \left(\infty ,t\right)=\theta_{\infty }\; \end{array}\}$$

Using the following transformation and thermophysical properties of nanoparticles (can be seen in) [55], the dimensionless governing equation for velocity and temperature profile are obtained;

(20) $$u^{*}=\frac{u}{u_{0}},\;\;t^{*}=\frac{u_{0}^{2}}{\nu }t,\;\;\theta^{*}=\frac{\theta -\theta_{\infty }}{\theta_{w}-\theta_{\infty }},\;\lambda_{1}^{*}=\frac{u_{0}^{2}}{\nu }\lambda_{1},\;\;y^{*}=\frac{u_{0}y}{\nu },$$

(21) $$\begin{array}{c} \frac{\rho_{nf}}{\rho_{f}}=a_{1}=\left(1-\phi \right)+\phi \frac{\rho_{s}}{\rho_{f}},\;\frac{{\left(\rho \beta_{\theta }\right)}_{nf}}{{\left(\rho \beta_{\theta }\right)}_{f}}=a_{2}=\left(1-\phi \right)+\phi \left(\frac{{\left(\rho \beta_{T}\right)}_{s}}{{\left(\rho \beta_{T}\right)}_{f}}\right)\\ \frac{\mu_{nf}}{\mu_{f}}=a_{3}=\frac{1}{{\left(1-\phi \right)}^{2.5}},\;\frac{{\left(\rho C_{p}\right)}_{nf}}{{\left(\rho C_{p}\right)}_{f}}=a_{4}=\left(1-\phi \right)+\phi \left(\frac{{\left(\rho C_{p}\right)}_{s}}{{\left(\rho C_{p}\right)}_{f}}\right)\\ \frac{k_{nf}}{k_{f}}=a_{5}=\frac{k_{s}+2k_{f}-2\phi \left(k_{f}-k_{s}\right)}{k_{s}+2k_{f}+\phi \left(k_{f}-k_{s}\right)},\;\frac{{\left(\sigma \right)}_{nf}}{{\left(\sigma \right)}_{f}}=a_{6}=1+\frac{3\left(\frac{\sigma_{s}}{\sigma_{f}}-1\right)\phi }{\left(\frac{\sigma_{s}}{\sigma_{f}}-2\right)-\left(\frac{\sigma_{s}}{\sigma_{f}}-1\right)\phi } \end{array}\}$$

The dimensionless velocity and temperature profile of the problem is given, and after omitting (∗) notation for the sack of brevity of mathematical modeling.

(22) $$\left(1+\lambda_{1}^{\alpha }D_{t}^{\alpha }\right)\left(\frac{\partial u}{\partial t}\right)=b_{1}\left(\frac{\partial^{2}u}{\partial y^{2}}\right)+b_{2}Gr\left(1+\lambda_{1}^{\alpha }D_{t}^{\alpha }\right)\left(\theta \right)-b_{3}H_{a}^{2}\left(1+\lambda_{1}^{\alpha }D_{t}^{\alpha }\right)\left(u\right).$$

Moreover, the temperature equation takes the following form

(23) $$b_{4}Pr\left(1+\lambda_{2}^{\beta }\;D_{t}^{\beta }\right)\left(\frac{\partial \theta }{\partial t}\right)=\left(1+Nr\right)\left(1+\lambda_{2}^{\beta }\;D_{t}^{\beta }\right)\frac{\partial^{2}\theta }{\partial y^{2}}+\left(1+\lambda_{2}^{\beta }\;D_{t}^{\beta }\right)Q_{0}\theta .$$

In Equations (22) and (23) $b_{1},\;b_{2},\;b_{3}\;\mathrm{and}\;b_{4}$ are the ratio of thermophysical properties given by

$$b_{1}=\frac{a_{3}}{a_{1}},\;b_{2}=\frac{a_{2}}{a_{1}},\;b_{3}=\frac{a_{6}}{a_{1}}\;\mathrm{and}\;b_{4}=\frac{a_{4}}{a_{5}}.$$

Additionally, $Gr,\;Ha,\;Pr,\;Nr\;\mathrm{and}\;Q_{0}$ are the Grashof number, Hartmann number, Prandtl number, radiation parameter and heat generation parameter, respectively, defined in [45] as;

$$Gr=\frac{\nu g{\left(\beta_{\theta }\right)}_{f}\left(\theta_{w}-\theta_{\infty }\right)}{u_{0}^{3}},H_{a}^{2}=\frac{\sigma_{f}B_{0}^{2}\nu }{\rho_{f}u_{0}^{2}},Pr=\frac{\mu {\left(C_{p}\right)}_{f}}{K_{f}},\;Nr=\frac{16\sigma^{*}\theta_{\infty }^{3}}{a_{5}3k^{*}K_{f}}\;\mathrm{and}\;Q_{0}=\frac{Q\nu^{2}}{a_{5}K_{f}u_{0}^{2}}.$$

Additionally, the non-dimensional initial and boundary conditions are

(24) $$u\left(y,0\right)=0,\;u_{t}\left(y,0\right)=0,\;u\left(0,t\;\right)=e^{at},\;u\left(\infty ,t\right)=0.$$

(25) θ(y,0)=0, θ(0,t)=1, θ(∞,t)=0.

The non-dimensional governing equation for velocity and temperature profile in Equations (22) and (23), with dimensionless initial and boundary conditions in Equations (24) and (25), represents the unsteady, incompressible flow fractional Maxwell nanofluids phenomena under the influence of magnetic fields. Water is taken as base fluid, but $Cu\;\mathrm{or}\;Al_{2}O_{3}$ are the nanoparticles considered for nanofluid preparation. For the numerical results the following Table 1 containing thermo-physical properties of nanoparticles and base fluid will be utilized.

## 3. Numerical Procedure

The finite difference scheme is a very efficient and powerful tool to investigate the numerical solutions of the problem arising and mathematical physics and mechanics. In this context, this section is dedicated to extending the finite-difference scheme to tackle the obtained set of fractional-order fluid models and heat transfer. For this, the discretization of the derivative of fractional-order of $u,\;u_{t}$ and $u_{yy}$ are specified as,

(26) D0Ctj+1αu(yi,tj+1)=Δt−αΓ(2−α)[uij+1−uij]+Δt−αΓ(2−α)∑l=1j(uij−l+1−uij−l)dlα,

(27) D0Ctj+11+αu(yi,tj+1)=Δt−(1+α)Γ(2−α)[uij+1−2uij+uij−1]+Δt−(1+α)Γ(2−α)×∑l=1j(uij−l+1−2uij−l+uij−l−1)dlα,

(28) $${\frac{\partial }{\partial t}u\left(y_{i},t_{j+1}\right)|}_{t=t_{j+1}}=\frac{1}{\Delta t}\left[u_{i}^{j+1}-u_{i}^{j}\right],$$

(29) $${\frac{\partial^{2}}{\partial y^{2}}u\left(y_{i+1},t_{j}\right)|}_{y=y_{i+1}}=\frac{1}{\Delta y^{2}}\left[u_{i+1}^{j+1}-2u_{i}^{j+1}+u_{i-1}^{j+1}\right].$$

and the nonlinear term is approximated by means of the following concept

$$u^{2}\left(y_{i},t_{j}\right)=u\left(y_{i},t_{j+1}\right)u\left(y_{i},t_{j}\right).$$

where, $d_{l}^{\alpha }=-l^{1-\alpha }+{\left(1+l\right)}^{1-\alpha }$ for l=1,2,3,…,j. Now, the rectilinear grid is assumed to examine the solution of the governing set of fractional-order fluid problems and heat transfer having grid spacing Δy>0, Δt>0 in the direction of space and time separately, where $\Delta t=\frac{T}{N},\;\Delta y=\frac{L}{M}$ for Δy, Δt from ${\mathbb{Z}}^{+}$. The inner points $\left(y_{i},t_{j}\right)$ in the discussed domain Ω=[0,T]×[0,L] are given as $i\Delta y=y_{i}$ and $j\Delta t=t_{j}$. The discretization of the governing set of fractional-order fluid problems and heat transfer at $\left(y_{i},t_{j}\right)$ is given as,

$$\begin{array}{c} \frac{1}{2\Delta t}\left(u_{i}^{j+1}-u_{i}^{j-1}\right)+\frac{\lambda_{1}^{\alpha }\Delta t^{-\left(1+\alpha \right)}}{\Gamma \left(2-\alpha \right)}\left(u_{i}^{j+1}-2u_{i}^{j}+u_{i}^{j-1}\right)+\frac{\lambda_{1}^{\alpha }\Delta t^{-\left(1+\alpha \right)}}{\Gamma \left(2-\alpha \right)}\\ \times \sum_{l=1}^{j}\left(u_{i}^{j-l+1}-2u_{i}^{j-l}+u_{i}^{j-l-1}\right)b_{l}^{\alpha }=\frac{b_{1}}{\Delta y^{2}}\left(u_{i+1}^{j+1}-2u_{i}^{j+1}+u_{i-1}^{j+1}\right)+b_{2}Gr\theta_{i}^{j+1}\\ +\frac{b_{2}\lambda_{1}^{\alpha }Gr\Delta t^{-\alpha }}{2\Gamma \left(2-\alpha \right)}\left(\theta_{i}^{j+1}-\theta_{i}^{j-1}\right)+\frac{b_{2}Gr\lambda_{1}^{\alpha }\Delta t^{-\alpha }}{2\Gamma \left(2-\alpha \right)}\sum_{l=1}^{j}\left(\theta_{i}^{j-l+1}-\theta_{i}^{j-l-1}\right)b_{l}^{\alpha }-b_{3}H_{a}^{2}u_{i}^{j+1}\\ -\frac{b_{3}H_{a}^{2}\lambda_{1}^{\alpha }\Delta t^{-\alpha }}{\Gamma \left(2-\alpha \right)}\left(u_{i}^{j+1}-u_{i}^{j}\right)+\frac{b_{3}H_{a}^{2}\lambda_{1}^{\alpha }\Delta t^{-\alpha }}{\Gamma \left(2-\alpha \right)}\sum_{l=1}^{j}\left(u_{i}^{j-l+1}-u_{i}^{j-l}\right)b_{l}^{\alpha },\\ \frac{b_{4}\mathrm{Pr}}{\Delta t}\left(\theta_{i}^{j+1}-\theta_{i}^{j}\right)+\frac{b_{4}\mathrm{Pr}\lambda_{2}^{\beta }\Delta t^{-\left(1+\beta \right)}}{\Gamma \left(2-\beta \right)}\left(\theta_{i}^{j+1}-2\theta_{i}^{j}+\theta_{i}^{j-1}\right)\\ +\frac{b_{4}\mathrm{Pr}\lambda_{2}^{\beta }\Delta t^{-\left(1+\beta \right)}}{\Gamma \left(2-\beta \right)}\sum_{l=1}^{j}\left(\theta_{i}^{j-l+1}-2\theta_{i}^{j-l}+\theta_{i}^{j-l-1}\right)b_{l}^{\beta }=\frac{1+\mathrm{Nr}}{\Delta y^{2}}\left(\theta_{i+1}^{j+1}-2\theta_{i}^{j+1}+\theta_{i-1}^{j+1}\right)\\ +\frac{\left(1+\mathrm{Nr}\right)\lambda_{2}^{\beta }\Delta t^{-\beta }}{\Delta y^{2}\Gamma \left(2-\beta \right)}\sum_{l=0}^{j}\left(\theta_{i+1}^{j-l+1}-2\theta_{i+1}^{j-l}+\theta_{i+1}^{j-l-1}\right)b_{l}^{\beta }-\frac{\left(1+\mathrm{Nr}\right)2\lambda_{2}^{\beta }\Delta t^{-\beta }}{\Delta y^{2}\Gamma \left(2-\beta \right)}\\ \times \sum_{l=0}^{j}\left(\theta_{i}^{j-l+1}-2\theta_{i}^{j-l}+\theta_{i}^{j-l-1}\right)b_{l}^{\beta }+\frac{\left(1+\mathrm{Nr}\right)\lambda_{2}^{\beta }\Delta t^{-\beta }}{\Delta y^{2}\Gamma \left(2-\beta \right)}\sum_{l=0}^{j}\left(\theta_{i-1}^{j-l+1}-2\theta_{i-1}^{j-l}+\theta_{i-1}^{j-l-1}\right)b_{l}^{\beta }\\ +\frac{Q_{0}\Delta t^{-\beta }}{\Gamma \left(2-\beta \right)}\left(\theta_{i}^{j+1}-\theta_{i}^{j}\right)+\frac{Q_{0}\Delta t^{-\beta }}{\Gamma \left(2-\beta \right)}\sum_{l=1}^{j}\left(\theta_{i}^{j-l+1}-\theta_{i}^{j-l}\right)b_{l}^{\beta }+Q_{0}\theta_{i}^{j+1}. \end{array}$$

for j=1,2,3,…,N−1, i=1,2,3,…,N−1, with the following initial and boundary conditions,

$$u_{i}^{0}=0,\;u_{i}^{1}=u_{i}^{-1},\;\theta_{i}^{0}=0,\;\theta_{i}^{1}=\theta_{i}^{-1},\;\mathrm{for}\;i=0,1,2,3,\ldots ,M,$$

$$u_{0}^{j}=\exp\left(aj\Delta t\right),\;u_{M}^{j}=0,\;\theta_{0}^{j}=1,\;\theta_{M}^{j}=0,\;\mathrm{for}\;j=1,2,3,\ldots ,N-1.$$

### 3.1. Numerical Analysis and Discussion

**Test Problem.** For the validation of the applied scheme, a test problem is considered as

D0Ctαu(y,t)=∂2∂y2u(y,t)−∂∂yu(y,t)+h(y,t)

In the discussed problem, the conditions are given below, and the source term can be selected against the choice of fractional-order derivative.

$$u\left(y,0\right)=u_{t}\left(y,t\right)=u\left(\infty ,t\right)=0\;\mathrm{and}\;u\left(0,t\;\right)=e^{at}$$

**Figure 2:**
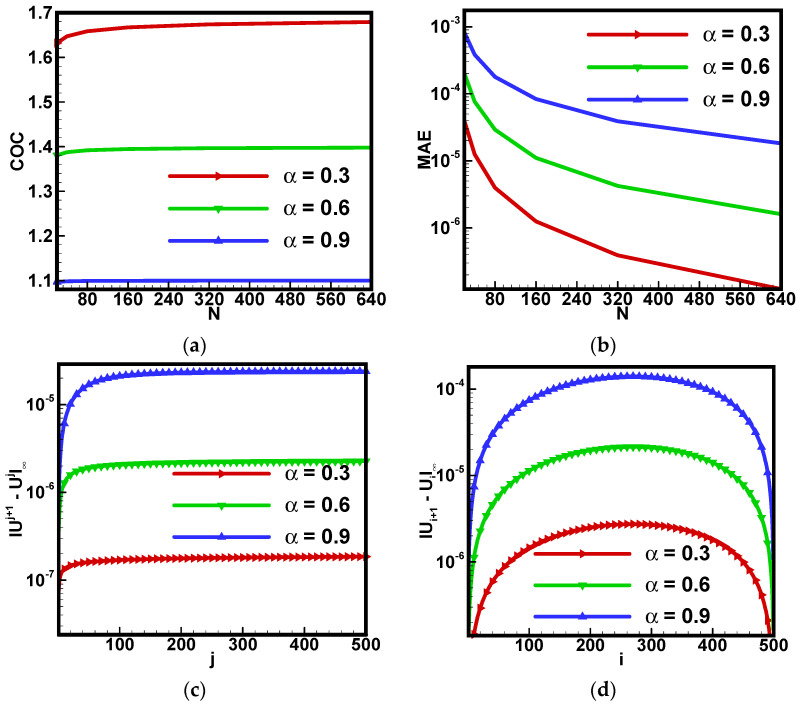
Code validation of proposed scheme and varying time mesh size against (**a**) computational order of convergence (COC) (**b**) maximum absolute error (MAE), and varying mesh size for (**c**) time and (**d**) space against *L*_∞_-norm between consecutive solutions.

Since such a physical problem contains $u\left(y,t\right)=y\left(y-t\right)t^{2}$ as the exact solution. Its accuracy has been checked by a number of simulations for the proposed scheme. The plots in Figure 2a,b are drawn for the maximum absolute error (MAE) and computational order of convergence (COC) for different ranges of *N,* which is *N* = 10, 20, 40, 80, 160, 320, 640.

$$\mathrm{MAE}=\underset{\begin{array}{c} 1\leq i\leq M\\ 1\leq j\leq N \end{array}}{\max}\left|u\left(y_{i},t_{j}\right)-u_{i}^{j}\right|,\;\mathrm{COC}=\log\left(\frac{\mathrm{MAE}\left(k\right)}{\mathrm{MAE}\left(k+1\right)}\right)/\log\left(\frac{N\left(k+1\right)}{N\left(k\right)}\right).$$

The convergence of the applied scheme is observed against the selection of each fractional-order derivative, and its convergence order enhances for α→1. Figure 2c,d contains the *L*_∞_-norm between consecutive solutions that is ${\left|u^{j+1}-u^{j}\right|}_{\infty }$ and ${\left|u_{i+1}-u_{i}\right|}_{\infty }$ when 0 ≤ *i, j* ≤ *N*, *M* = 500. Again, it is found that the proposed scheme is very efficient, accurate and reliable for this problem. It also demonstrates that the solution is stable against the selection of fractional order and mesh parameters.

### 3.2. Entropy Generation

For viscous fluid flow in a magnetic field, the volumetric rate of local entropy generation $E_{G}$ is defined in [57] as;

(30) $$E_{G}=E_{\theta }+E_{f}+E_{m},$$

That is the sum of entropy generation due to heat transfer, due to fluids friction and due to the magnetic field effect, separately mentioned here (can be seen in [33])

$$E_{\theta }=\frac{K_{nf}}{\theta_{\infty }^{2}}\left(1+\frac{16\sigma^{*}\theta_{\infty }^{3}}{3k^{*}K_{nf}}\right){\left(\frac{\partial \theta }{\partial y}\right)}^{2},\;E_{f}=\frac{\mu_{nf}}{\theta_{\infty }}{\left(\frac{\partial u}{\partial y}\right)}^{2}\mathrm{and}\;E_{m}=\frac{\sigma_{nf}B_{0}^{2}}{\theta_{\infty }}u^{2}$$

Combining all the results

(31) $$E_{G}=\frac{K_{nf}}{\theta_{\infty }^{2}}\left(1+\frac{16\sigma^{*}\theta_{\infty }^{3}}{3k^{*}K_{nf}}\right){\left(\frac{\partial \theta }{\partial y}\right)}^{2}+\frac{\mu_{nf}}{\theta_{\infty }}{\left(\frac{\partial u}{\partial y}\right)}^{2}+\frac{\sigma_{nf}B_{0}^{2}}{\theta_{\infty }}u^{2},$$

The dimensionless entropy generation calculated is;

(32) $$N_{S}=\frac{E_{G}}{E_{0}}=\left[a_{5}\left(1+N_{r}\right){\left(\frac{\partial \theta }{\partial y}\right)}^{2}+a_{3}\frac{Br}{\Omega }{\left(\frac{\partial u}{\partial y}\right)}^{2}+a_{6}\frac{Br}{\Omega }H_{a}u^{2}\right]$$

where $E_{0}=\frac{K_{f}}{\nu^{2}}\frac{u_{0}^{2}{\left(\theta_{w}-\theta_{\infty }\right)}^{2}}{\theta_{\infty }^{2}},\;\Omega =\frac{\left(\theta_{w}-\theta_{\infty }\right)}{\theta_{\infty }},Br=\frac{\mu_{f}u_{0}^{2}\;}{K_{f}\left(\theta_{w}-\theta_{\infty }\right)},H_{a}=M=\frac{\sigma_{f}B_{0}^{2}\nu }{\rho_{f}u_{0}^{2}}\;\mathrm{and}\;N_{r}=\frac{16\sigma^{*}\theta_{\infty }^{3}}{a_{5}3k^{*}K_{f}}.$
where $E_{0}$, is the characteristic entropy generation rate, Br is the Brickman number, $H_{a}^{2}=M$ is the square of the Hartmann Number and Ω is the dimensionless temperature difference.

#### Bejan Number

The Bejan number is the irreversibility distribution parameter which is expressed mathematically as (can be seen in [58]);

$$Be=\frac{\mathrm{Entropy}\;\mathrm{generation}\;\mathrm{due}\;\mathrm{heat}\;\mathrm{transfer}}{\mathrm{Total}\;\mathrm{Entropy}\;\mathrm{generation}},$$

That is

(33) $$Be=\frac{\frac{K_{f}u_{0}^{2}{\left(\theta_{w}-\theta_{\infty }\right)}^{2}}{\nu^{2}\theta_{\infty }^{2}}\left[a_{5}\left(1+N_{r}\right){\left(\frac{\partial \theta }{\partial y}\right)}^{2}\right]}{\frac{K_{f}}{\nu^{2}}\frac{u_{0}^{2}{\left(\theta_{w}-\theta_{\infty }\right)}^{2}}{\theta_{\infty }^{2}}\left[a_{5}\left(1+N_{r}\right){\left(\frac{\partial \theta }{\partial y}\right)}^{2}+a_{3}\frac{Br}{\Omega }{\left(\frac{\partial u}{\partial y}\right)}^{2}+a_{6}\frac{Br}{\Omega }H_{a}^{2}{\left(u\right)}^{2}\right]}$$

Then the reduced expression for the Bejan number is given as;

(34) $$Be=\frac{\left[\left(1+N_{r}\right){\left(\frac{\partial \theta }{\partial y}\right)}^{2}\right]}{\left[\left(1+N_{r}\right){\left(\frac{\partial \theta }{\partial y}\right)}^{2}+b_{5}\frac{Br}{\Omega }{\left(\frac{\partial u}{\partial y}\right)}^{2}+b_{6}\frac{Br}{\Omega }H_{a}^{2}{\left(u\right)}^{2}\right]}.$$

With $b_{5}=\frac{a_{3}}{a_{5}},\;b_{6}=\frac{a_{6}}{a_{5}}.$

### 3.3. Skin Friction and Nusselt Number

For measuring shear stress and heat transfer effects in an ordinary integer order system, local skin friction and Nusselt number are defined in [59] as;

(35) $$S_{f}=-\mu_{nf}{\left(\frac{\partial u}{\partial y}\right)}_{y=0}.$$

and

(36) $$Nu=-K_{nf}\left(1+\frac{16\sigma^{*}\theta_{\infty }^{3}}{3k^{*}K_{f}}\right){\left(\frac{\partial \theta }{\partial y}\right)}_{y=0}$$

The skin friction coefficient and local Nusselt number for (FMF) can be written by using Equation (5), that is, the fractional stress tensor for Maxwell fluid on the plate with fractional time Caputo derivative (detail can be seen in [60]).

(37) $$S_{f}+\lambda_{1}^{\alpha }\frac{\partial^{\alpha }S_{f}}{\partial t^{\alpha }}=-\mu_{nf}{\left(\frac{\partial u}{\partial y}\right)}_{y=0}.$$

(38) $$Nu+\lambda_{1}^{\beta }\frac{\partial^{\beta }Nu}{\partial t^{\beta }}=-K_{nf}\left(1+\frac{16\sigma^{*}\theta_{\infty }^{3}}{3k^{*}K_{f}}\right){\left(\frac{\partial \theta }{\partial y}\right)}_{y=0}.$$

The non-dimensional form of Equations (38) and (39) is given as

(39) $$S_{f}+\lambda_{1}^{\alpha }\frac{\partial^{\alpha }S_{f}}{\partial t^{\alpha }}=-a_{3}\mu_{f}{\left(\frac{\partial u}{\partial y}\right)}_{y=0}.$$

(40) $$Nu+\lambda_{1}^{\beta }\frac{\partial^{\beta }Nu}{\partial t^{\beta }}=-a_{5}\left(1+Nr\right){\left(\frac{\partial \theta }{\partial y}\right)}_{y=0}.$$

## 4. Results and Discussion

This section of the article deliberates the detailed results and discusses the plots driven against different physical parameters like $H_{a},\;Pr,\;Nr,\;Gr,\;\phi ,\;\alpha ,\;\beta \;\mathrm{and}\;Q_{0}$ representing the magnetic field parameter (the square of Hartmann Number), Prandtl number, radiation parameter, Grashof Number, volumetric fraction of nanoparticle, fractional order parameters and heat generation parameter, respectively. The behavior of these aforementioned physical parameters on dimensionless velocity profile u(y,t), temperature profile θ(y,t), Bejan number-BN Be, Skin friction $S_{f}$ and Nusselt number Nu are drawn by mathematical software MAPLE. The mathematical fractional model of Maxwell nanofluid is developed by using the Caputo time fractional operator. After transforming the governing equations to a dimensionless governing model, the finite difference method (FDM) is used for the discretization of the model. FDM is a strong tool for dealing with such kinds of problems. The simulation is performed by developing and executing codes.

Results are obtained by solving Equations (22), (23), (32), (39) and (40) with initial and boundary conditions illustrated in Equations (24) and (25) and physical properties of nanoparticles in Equation (21) and Table 1. Various suitable ranges of physical paramet $\left(H_{a}=1,\;2,\;5\right),\;\left(Pr=3.5,\;6.2,\;15\right),\;\left(Gr=0,\;1,\;2\right),\;\left(\phi =0.01,\;0.1,0.2\right),\;\left(\alpha =0.2,\;0.4,\;0.6,\;0.8,\;1\right),\;\left(Nr=0,\;2,\;5\right),\;\left(Q_{0}=0,\;2,\;5\right)$ for heat transfer, velocity analysis, skin friction, Nusselt number, entropy generation and Bejan number—BN are considered, and also particular exertion has been done on the effects of these parameters for heat enhancement.

**Figure 3:**
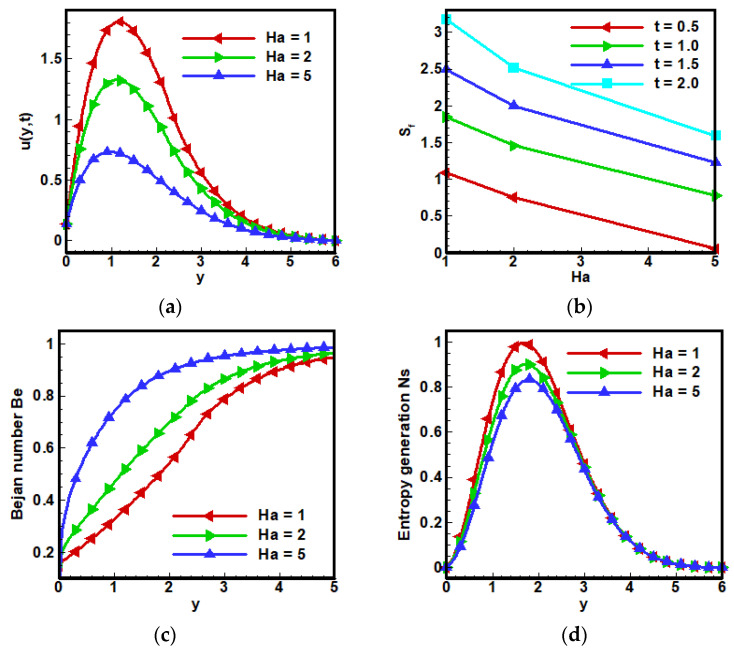
Impact of the Hartmann number on (**a**) dimensionless velocity profile, (**b**) coefficient of skin friction, (**c**) Bejan number and (**d**) entropy generation when $\Lambda_{1}=0.6,\;\Lambda_{2}=0.5,\;Gr=5,\;Ha=10,\mathrm{Pr}=6,\;Q_{0}=5,Nr=3.5,a=-1,\;Br=0.5,\;\Omega =10,\;\phi =0.1$.

Figure 3 shows the effect of the magnetic field parameter on the velocity profile of fractional Maxwell fluids. It can be seen that velocity decreased by increasing the value of $H_{a}$ because of the Lorentz’s force. The rise in $H_{a}$ caused strengthening in the Lorentz force, which increases the internal resistance to flow particles; consequently, fluid velocity decreased. Whereas an increase in Skin friction occurs, as shown in Figure 3b. Since Bejan number Be is the ratio of total entropy generation to entropy generation due to heat transfer. Thus, advancement in $H_{a}$ boosts the Bejan number-BN Be and can be noticed in Figure 3c. An opposite behavior of total entropy generation $N_{S}$ relative to Bejan number—BN Be appears against the increasing value of $H_{a}$, as can be seen in Figure 3d.

**Figure 4:**
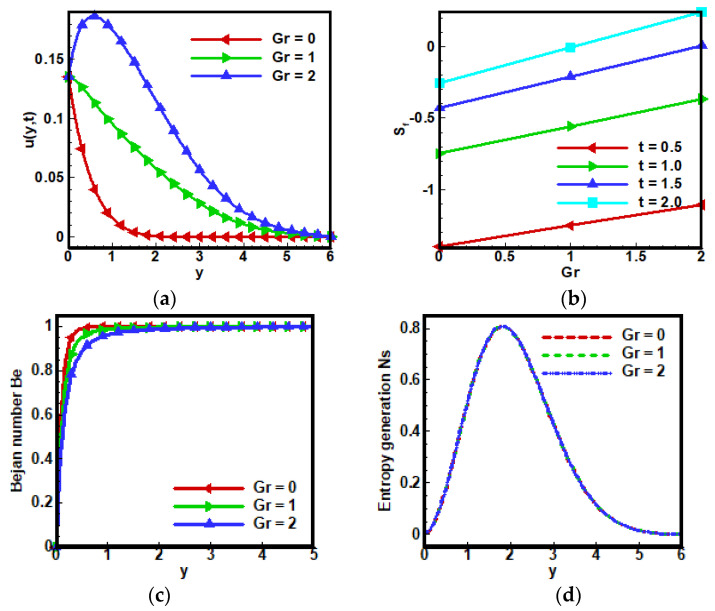
Influence of Grashof number Gr on (**a**) dimensionless velocity profile, (**b**) coefficient of skin friction, (**c**) Bejan number and (**d**) entropy generation when $\Lambda_{1}=0.6,\;\Lambda_{2}=0.5,\;Gr=5,\;Ha=10,\mathrm{Pr}=6,\;Q_{0}=5,Nr=3.5,a=-1,\;Br=0.5,\;\Omega =10,\;\phi =0.1$.

Figure 4 depicts the effects of the Grashof number Gr on velocity profile u(y,t). The enhancement in value of Gr results in increasing the fluid velocity, which can be physically justified as the increasing value of Gr means lower the viscous forces and hence increasing the velocity of fractional Maxwell fluids. While Skin friction $S_{f}$ increases, the increasing the value of Gr can be noticed in Figure 4b. A slight effect of Gr on Bejan number—BN Be and entropy generation $N_{s}$ can be seen in Figure 4c,d, respectively. Similar results are reported by Sarojamma, G., et al. in [61] for comparison. Gr is the ratio of buoyancy forces to viscous force, increasing value of Gr results in laminar boundary layer and vice versa. Because high value of Gr give rise to the temperature of molecules, consequently, the intermolecular forces become weak. Thus, velocity profile u(y,t) also rises. On the other hand, fluid particles collectively gain momentum as Gr increases, so additional heat is lost nearby, that is why skin friction reduces as shown in Figure 4b. Since Bejan number Be is ratio of entropy generation due to heat to the total entropy generation of the system, that is why Gr reduces the value of Bejan number—BN Be deliberated in Figure 4c.

**Figure 5:**
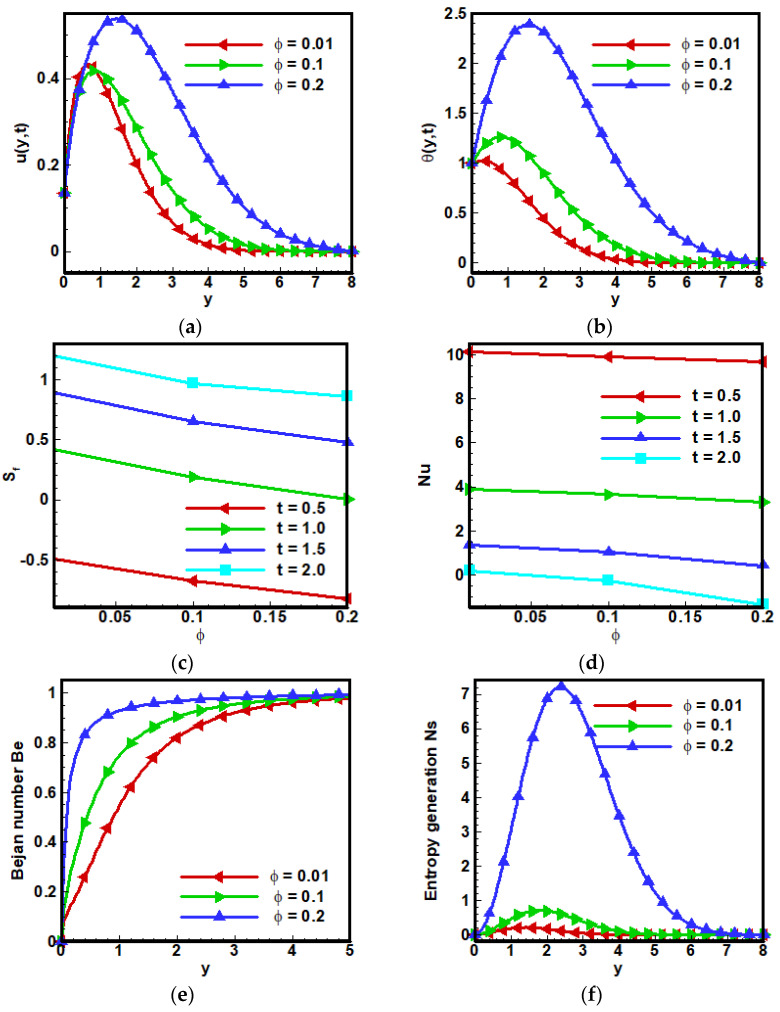
Effect of ϕ on (**a**) dimensionless velocity profile, (**b**) non-dimensional temperature profile, (**c**) coefficient of skin friction, (**d**) local Nusselt number Nu, (**e**) Bejan number and (**f**) entropy generation when $\Lambda_{1}=0.6,\;\Lambda_{2}=0.5,\;Gr=5,\;Ha=10,\mathrm{Pr}=6,\;Q_{0}=5,Nr=3.5,a=-1,\;Br=0.5,\;\Omega =10,\;\phi =0.1$.

Figure 5 shows the effects of the volume fraction parameter ϕ on fluid velocity, and it is examined that advancement in controlled volume fraction parameter ϕ lowers the velocity profile due to the effect; that is, the viscosity of fractional Maxwell fluids increases by increasing ϕ. On the other hand, the addition of nanoparticles in the base fluid causes an improvement in the heat transfer rate at the boundary layer. As the thermal conductivity of base fluid is enhanced, consequently increasing the fluid’s internal temperature can be seen in Figure 5b. Since Nusselt number Nu is the ratio of convective heat transfer to conductive heat transfer, increasing ϕ has a decreasing relation with Nusselt number Nu. This is because the skin friction decreases with the passage of time against the volume fraction parameter ϕ. These results for skin friction and Nusselt number Nu are viewed in Figure 5c,d, respectively. An increasing trend is noticed for the Bejan number Be and can be seen in Figure 5e as the heat transfer rate becoming better by increasing ϕ. Whereas in Figure 4f entropy generation $N_{s}$ enhanced by increasing the value of ϕ. Because the viscosity of fluid increased by increasing ϕ.

**Figure 6:**
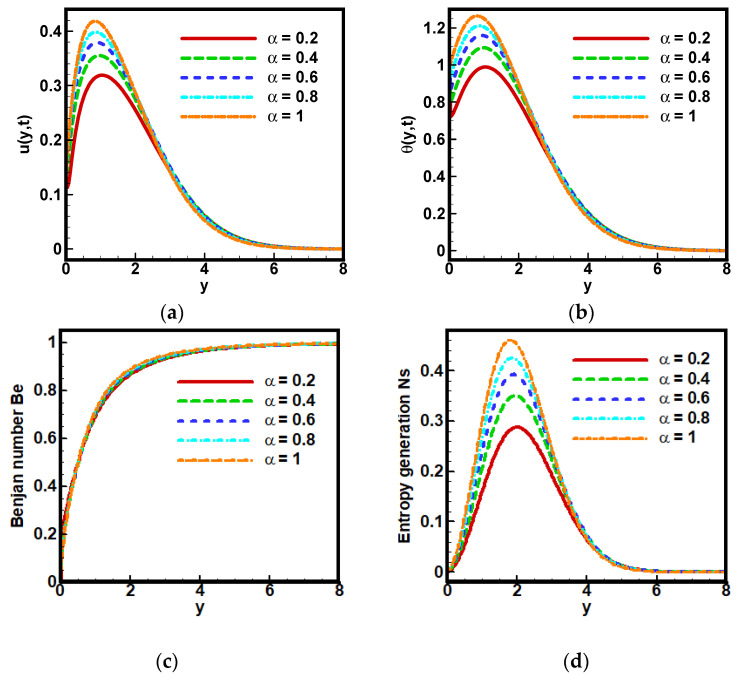
Impact of fractional parameter α=β on (**a**) dimensionless velocity profile, (**b**) non-dimensional temperature profile, (**c**) Bejan number and (**d**) Entropy generation when $\Lambda_{1}=0.6,\;\Lambda_{2}=0.5,\;Gr=5,\;Ha=10,\mathrm{Pr}=6,\;Q_{0}=5,Nr=3.5,a=-1,\;Br=0.5,\;\Omega =10,\;\phi =0.1$.

In these following plots in Figure 6, results are drawn for velocity profile u(y,t), temperature profile θ(y,t), skin friction $S_{f}$, Nusselt number Nu and Bejan number-BN Be against fractional parameter α.

The reason is that the gradual increase in fractional parameter α gives rise to the viscosity of the nanofluid. This means intermolecular forces between the nanoparticles and base-fluid particles increase; consequently, Brownian motion of particles reduces, that is, a decrease in velocity profile u(y,t) occurs. The consequences of fractional order parameter α for velocity profile u(y,t) are inverse as depicted in Figure 6a. On the other hand, α have direct relation for θ(y,t), as shown in Figure 6b. Figure 6c shows that for an increasing value of α the entropy generation $N_{s}$ decreases, whereas a rise in α varies Be directly, deliberated in Figure 6d.

**Figure 7:**
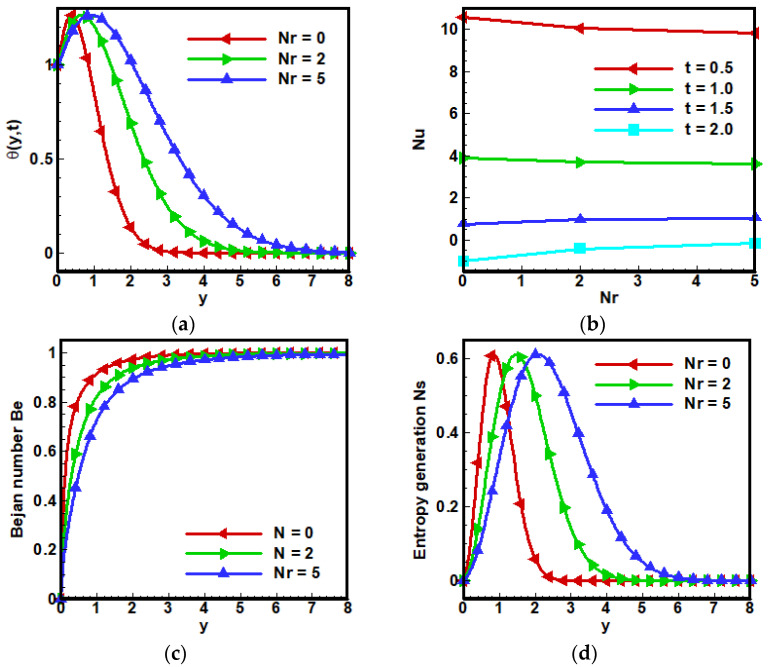
Influence of thermal radiation parameter Nr on (**a**) dimensionless velocity profile, (**b**) non-dimensional temperature profile, (**c**) Bejan number and (**d**) entropy generation when $\Lambda_{1}=0.6,\;\Lambda_{2}=0.5,\;Gr=5,\;Ha=10,\mathrm{Pr}=6,\;Q_{0}=5,Nr=3.5,a=-1,\;Br=0.5,\;\Omega =10,\;\phi =0.1$.

Figure 7 shows the effects of thermal radiation parameter $N_{r}$ on temperature θ(y,t) of the fluid. Applying thermal radiations gives rise to the temperature of particles of nanofluids. Hence, the particles’ kinetic energy increases, and the rate of collision between the particles of the nanofluids becomes high, which is why a rising increase in temperature profile occurs. Therefore it is concluded that increasing the value of $N_{r}$ causes an increase in fluid temperature. Nu is the ratio of convective to conductive heat transfer across the boundary, but enhancement in conduction occurs with the addition of nanoparticle therefore, a decrease occurs in Nu, depicted in Figure 7a,b, respectively. A decrease in Bejan number—BN Be can be noticed in Figure 7c, whereas entropy decreases initially and then increases gradually, as can be seen in Figure 7d. Similar results are reported in [61].

**Figure 8:**
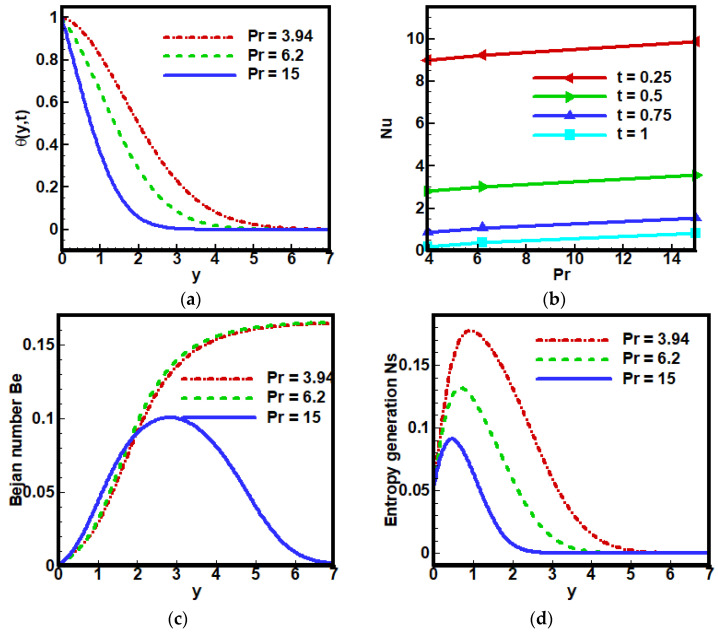
Effect of Pr on (**a**) dimensionless velocity profile, (**b**) coefficient of skin friction, (**c**) Bejan number and (**d**) entropy generation, when $\Lambda_{1}=0.1,\;\Lambda_{2}=0.2,\;Gr=5,\;Ha=2,\mathrm{Pr}=6.2,\;Q_{0}=2.5,Nr=5,\;Br=2,\;\Omega =10,\;\phi =0.1$.

Prandtl number Pr is the dimensionless number and is the ratio of momentum to thermal diffusivity. It is a fluid property but does not have any dependence on flow type. Thus, an increase in Pr means heat transfer is favored to occur by momentum, not conduction. This parameter controls the relative thickness and thermal boundary layer in heat transfer problems. Lowering the value of Pr means the heat diffuses spontaneously as compared to momentum, which thickens the thermal boundary layer rather than the momentum boundary layer. Therefore, an increase in Pr decreases the temperature profile θ(y, t) of fractional Maxwell fluids as expressed in Figure 8a, which expectedly decreases the Nusselt number Nu. Since Bejan number Be has an inverse relation with entropy generation $N_{s}$, due to heat transfer, that is, Be decreases with the increase in value of Pr as illustrated in Figure 8.

**Figure 9:**
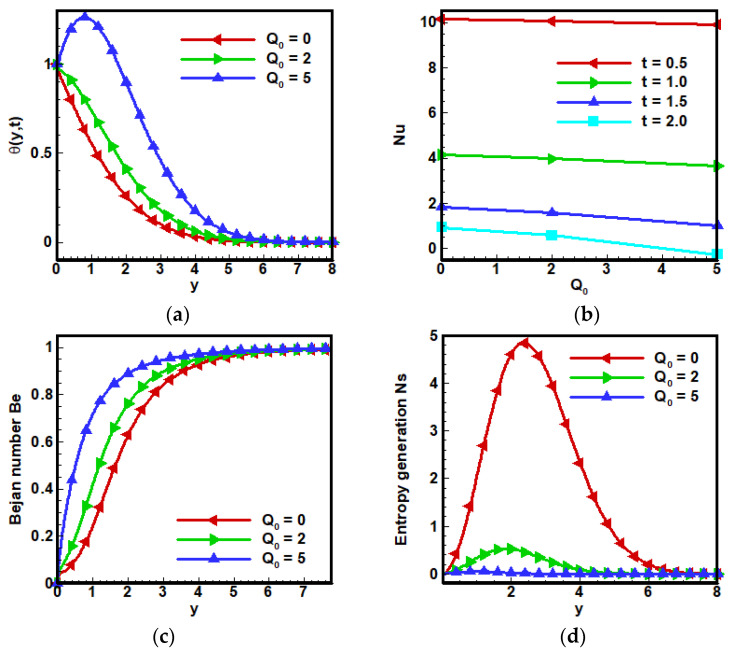
Influence of heat source $Q_{0}$ on (**a**) dimensionless velocity profile (**b**) non-dimensional temperature profile (**c**) Bejan number and (**d**) entropy generation when $\Lambda_{1}=0.6,\;\Lambda_{2}=0.5,\;Gr=5,\;Ha=10,\mathrm{Pr}=6,\;Q_{0}=5,Nr=3.5,a=-1,\;Br=0.5,\;\Omega =10,\;\phi =0.1$.

Figure 9 shows the effects of the heat source term $Q_{0}$ on temperature profile θ(y,t), since heat source gives rise to temperature profile as shown in Figure 9a, but with the passage of time, it is noticed that the Nusselt number Nu decrease is dependent inversely on the conduction of heat. Thus, entropy generation $N_{s}$ expectedly decreases as shown in Figure 9d. This phenomenon gives rise to the Bejan number Be because entropy generation due to heat transfer has an inverse relation with the Bejan number Be, as shown in Figure 9c.

## 5. Conclusions

The graphical analysis of fractional Maxwell nanofluids is made in this article under the influence of a magnetic field (ignoring induced magnetic field). The pressure gradient is supposed to be absent. Effects of different physical parameters are drawn by using the mathematical software MAPLE. The model is formulated by applying the Caputo time derivative. Using suitable transformations, governing equations are made dimensionless.

MHD fractional Maxwell nanofluids are studied numerically; quantities like entropy generation, Bejan number, Skin friction, and Nusselt number are investigated using the finite difference method.

Hence key findings of this study are given below;

a- It is noted that for increasing the value of fractional order parameter α, the velocity profile u(y,t) decreases, whereas the temperature profile θ(y,t) increases.
b- The addition of nanoparticles to base fluid enhances the thermal conductivity of fractional Maxwell nanofluids, increasing the value of volume fraction of nanoparticles ϕ and decreasing entropy generation $N_{s}$.
c- The magnetic field effect influences the temperature θ(y,t) and velocity u(y,t) profile with inverse and direct behavior, respectively.
d- Nusselt number increases with the variation in Pr, and a decrease occurs in Nu with the increase in thermal radiation parameter.
e- The temperature profile varies directly with the thermal radiation parameter Nr, and increasing the value of Nr decreases the Nusselt number. Whereas entropy generation $N_{s}$ increases, and the Bejan number decrease with a rising value of Nr.

The solution obtained via the finite difference method is excellent in agreement with the test problem and existing results which shows that the finite difference method is a strong and reliable technique to deal with such kind of complex models and it gives a key direction for further study.

## Figures and Tables

**Table 1:** The thermophysical properties of different base fluids and nanoparticles at room 25 °C [56].

Material	$H_{2}O$	Cu	$Al_{2}O_{3}$
$\rho \left({\mathrm{kgm}}^{-3}\right)$	997	8933	3970
$C_{p}\left({J\;\mathrm{Kg}}^{-1}k^{-1}\right)$	4197	385	765
$k\left({\mathrm{Wm}}^{-1}k^{-1}\right)$	0.613	400	40
$\beta \times 10^{-5}\left(k^{-1}\right)$	21	1.67	0.85
$\sigma {\left(\Omega m\right)}^{-1}$	0.05	$5.96\times 10^{7}$	$2.6\times 10^{6}$

## Data Availability

Not applicable.
